# Surgical Management of Arteriovenous Malformation of the Penis in an Adolescent Boy: A Case Report and Review of the Literature

**DOI:** 10.7759/cureus.44698

**Published:** 2023-09-05

**Authors:** Chrysostomos Kepertis, Maria Florou, Vassileios Mouravas, Maria Tsopozidi, Ioannis Spyridakis

**Affiliations:** 1 2nd Department of Pediatric Surgery, Papageorgiou General Hospital, Thessaloniki, GRC; 2 2nd Department of Pediatric Surgery, Aristotle University of Thessaloniki, Papageorgiou General Hospital, Thessaloniki, GRC

**Keywords:** transplant tissue, surgical excision, glans penis, children, arteriovenous malformation

## Abstract

Arteriovenous malformations (AVMs) are common benign lesions, present at birth. Although they may occur anywhere in the body, usually they are found on the head, the neck, and the extremities. AVMs of the glans penis are very rare. Only a few have been reported in the literature, and thus, a definitive treatment does not exist. We herein report our experience of treating an AVM of the glans penis with surgical excision and plastic reconstruction of the glans, with the application of transplant tissue from the inner skin of the prepuce.

## Introduction

Arteriovenous malformations (AVMs) are common congenital anomalies that occur due to errors during fetal vasculogenesis. They are defined as persistent connections between arteries and veins, proximal to the normal capillary bed. They are most frequently located on the head, the neck, the limbs, the tract, and the viscera [1]. Although AVMs have been reported in the scrotum [2], there are very few reports of a penile location in the literature. Because of the rarity of this clinical condition, definitive management does not exist [1,3]. Here, we present a case of an AVM on the glans penis of an adolescent boy who was treated with surgical excision and plastic reconstruction of the glans, with the application of transplant tissue from the inner foreskin. The patient recovered smoothly, the cosmetic results are excellent, and there are no complications or functional sequelae in the long-term follow-up. The procedure was performed for the first time in our surgical department and to our knowledge this is the first report of a congenital, penile AVM treated with excision and graft. 

## Case presentation

**Figure 1:**
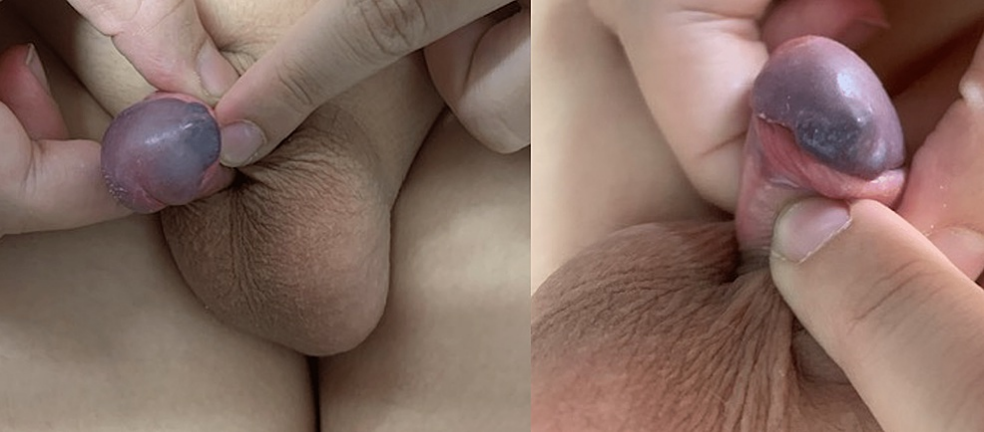
Preoperative clinical examination of the lesion on the glans penis.

A 12-year-old boy presented to our outpatient clinic, with a lesion on the glans penis. The lesion was present at birth and grew progressively and proportionally with the patient, without any symptoms such as pain, hematuria, or dysuria since then. The clinical examination revealed a well-shaped, soft, compressible, and non-pulsatile nodule on the left side of the penis (Figure 1).

**Figure 2:**
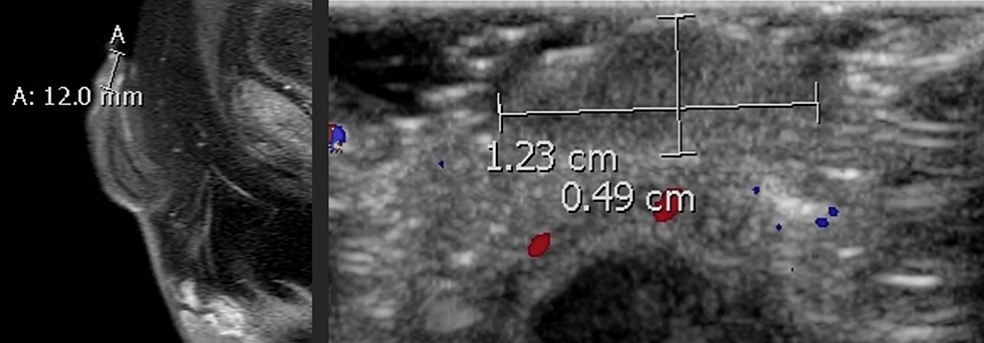
MRI and ultrasonography examinations of the lesion.

The nodule seemed like a vascular malformation and was non-pulsatile in physical examination. No history of trauma, hematuria, or urinary dysfunction was reported. The foreskin, the scrotal skin, and the rest of the penile skin were healthy, and the testes were of appropriate size, palpable in the scrotum. There were no other lesions on the body of the child. Personal history and family history were insignificant. Blood tests and urinalysis results were normal. Ultrasonography and magnetic resonance imaging of the lower abdomen and the pelvis revealed the presence of a 1.2 x 0.8 cm subepithelial lesion within the lamina propria on the left side of the glans penis, without any other abnormal findings (Figure 2). 

**Figure 3:**
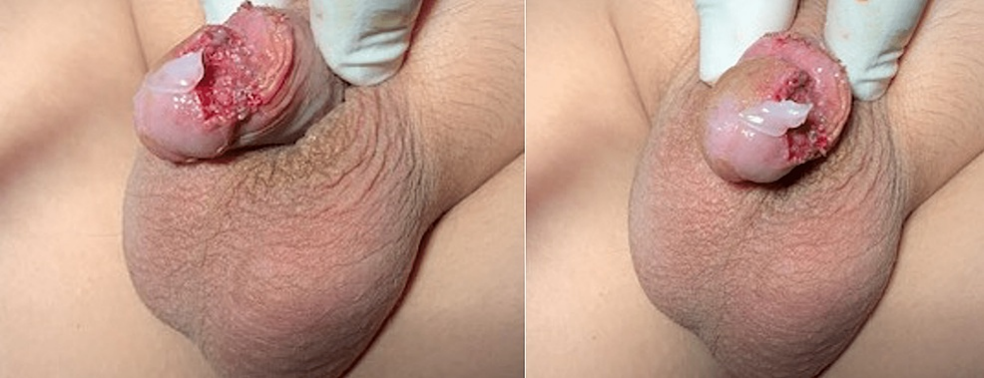
Cosmetic results after one week postoperatively.

**Figure 4:**
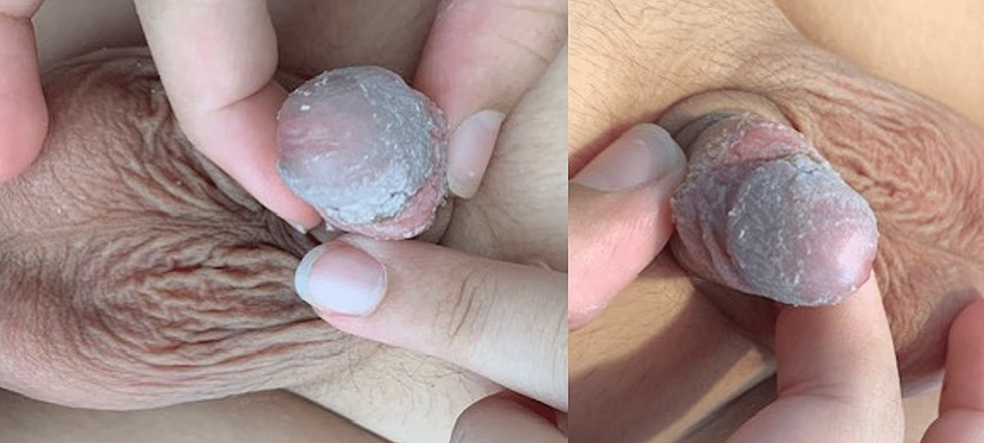
Cosmetic results after one month postoperatively.

**Figure 5:**
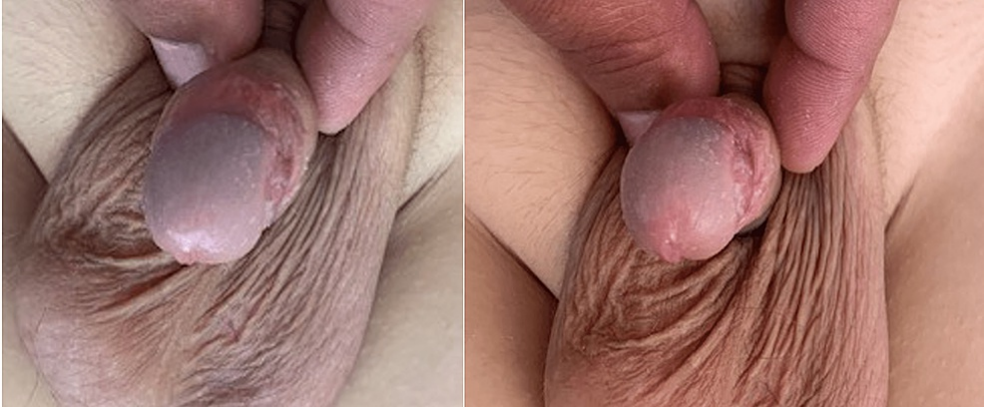
Cosmetic results after two months postoperatively.

The patient and the parents were informed about the treatment options: 1. Interventional ablation techniques, 2. Laser application methods, and 3. Surgical excision, and they decided to continue with the surgical management. At surgery, a urethra-cystoscopy was first applied with normal findings. The microsurgical excision of the vascular mass followed. The anatomical plane for the dissection was well-defined, as the lesion was found in the subepithelial tissue and totally separated from the corpus cavernosa and the spongiosum. After the excision, glansplasty was performed, with the application of a graft from the inner foreskin. There was a Foley catheter inserted in the urethra, in order to keep the area dry and clean from the urine postoperatively. The postoperative course was uneventful, the patient was on intravenous antibiotics and analgesia for two days, and the Foley catheter was removed on the second postoperative day. The patient was discharged home on the fourth postoperative day, and an antibiotic cream was applied over the glansplasty for the following three weeks. The histopathology findings made the diagnosis of an AVM of the glans penis. The follow-up did not show symptoms of residual tissue or recurrence, the cosmetic result was excellent, and the patient and his parents were completely satisfied (Figures 3-5).

## Discussion

This was the first case of an AVM on the glans penis treated with surgical excision and glansplasty in our department. AVMs are congenital vascular lesions, resulting from errors during fetal vasculogenesis. They consist of persistent connections between arteries and veins, adjacent to the normal capillary bed. AVMs are present at birth, grow progressively, and proportionally with the child, with the potential to respond to stimuli such as puberty hormones, stress, or surgery and they have a very low chance of spontaneous regression. They affect both boys and girls and they are most frequently located on the head, the neck, the limbs, the tract, and the viscera [1,4]. Although AVMs have been reported in the scrotum [2], there are very few reports of a penile location in the literature. According to their incidence, AVMs represent 8% of all congenital vascular anomalies [4], and only 1.7% of them are estimated to occur in the penis [5]. Clinical characteristics include a soft, compressible, non-pulsatile, and painless nodule, with potential to escalate to skin disfigurement, swelling, pain, ulceration, and bleeding [3,6]. Furthermore, in the literature, there are few reports of associated episodes of urethral bleeding and pain while urinating [6,7]. Because of the rarity of this clinical condition, definitive management does not exist [1,8]. The timing of treatment for a vascular lesion of the genitalia is different from other parts of the body. Since this anatomical area is covered, there is less social pressure for prompt management early in life. However, this changes dramatically when approaching adolescence and this clinical condition may cause great discomfort and anxiety to the young patient and his family [5,7]. It is advisable to postpone final management until structures are developed in size and shape, unless there are indications earlier in life that urge prompt treatment, such as bleeding, pain, and functional problems [5]. Various methods have been proposed for the management of AVMs [1]. The application of neodymium: yttrium-aluminum-garnet (Nd: YAG) laser has been introduced with good cosmetic results, although residual scarring, meatal retraction, and extended coagulation necrosis further than the target area have been highlighted [9,10]. The second management option is the sclerotherapy, that includes the intralesional injection of a sclerotic agent, usually bleomycin or ethanol [11]. Although some good results have been reported, there is a risk of nontargeted embolization, adjacent tissue damage, scarring, and ulceration [12,13]. Surgical excision had been the standard approach for all vascular malformations, with bleeding and scarring as the main two complications, before the development of less interventional methods. A meticulous surgical procedure is the core of a successful approach [14]. In the presented case report, the surgical excision was selected because the lesion was small and localized with defined borders. The preoperative imaging had indicated a clear anatomical plane and it was considered safe to proceed. The postoperative cosmetic results and the satisfaction of the patient verified the selected treatment plan. Last but not least, AVMs are a heterogenous clinical entity and some authors suggest a combination of treatment options, especially when it comes to the external genitalia [5].

## Conclusions

In conclusion, we treated a penile AVM with surgical excision of the lesion followed by plastic reconstruction of the glans, with the application of transplant tissue from the inner foreskin. The treatment of children affected by AVMs is still considered a challenge for pediatric surgeons, especially when they are located in delicate anatomic areas such as the external genitalia. AVMs are treated by sclerotherapy, embolization, and/or surgical excision. The patient’s satisfaction and the functional and cosmetic results define the treatment outcomes. Taking into account the rarity of this clinical entity, more long-term, large studies are needed in the future to confirm the current literature data and potentially indicate a specific treatment strategy.
